# The rs1127354 Polymorphism in *ITPA* Is Associated with Susceptibility to Infertility

**DOI:** 10.22074/cellj.2018.4255

**Published:** 2018-01-01

**Authors:** Fahimeh Mollaahmadi, Ashraf Moini, Reza Salman Yazdi, Mehrdad Behmanesh

**Affiliations:** 1 Department of Genetics, Faculty of Biological Sciences, Tarbiat Modares University, Tehran, Iran; 2 Department of Endocrinology and Female Infertility, Reproductive Biomedicine Research Center, Royan Institute for Reproductive Biomedicine, ACECR, Tehran, Iran; 3 Department of Andrology, Reproductive Biomedicine Research Center, Royan Institute for Reproductive Biomedicine, ACECR, Tehran, Iran

**Keywords:** Infertility, *ITPA*, Genotyping, Single Nucleotide Polymorphism

## Abstract

**Objective:**

Infertility is a common human disorder which is defined as the failure to conceive for a period of 12 months without contraception. Many studies have shown that the outcome of fertility could be affected by DNA damage. We attempted to examine the association of two SNPs (rs1127354 and rs7270101) in *ITPA*, a gene encoding a key factor in the repair system, with susceptibility to infertility.

**Materials and Methods:**

This was a case-control study of individuals with established infertility. Blood samples were obtained from 164 infertile patients and 180 ethnically matched fertile controls. Total genomic DNA were extracted from whole blood using the standard salting out method, and stored at -20˚C. Genotyping were based on mismatch polymerase chain reaction-restriction fragment length polymorphism (PCR-RFLP) method in which PCR products were digested with the XmnI restriction enzyme and run on a 12% polyacrylamide gel.

**Results:**

All genotype frequencies in the control group were in Hardy-Weinberg equilibrium. A significant association (in allelic, recessive and dominant genotypic models) was observed between infertile patients and healthy controls based on rs1127354 (P=0.0001), however, no significant association was detected for rs7270101. Also, gender stratification and analysis of different genotype models did not lead to a significant association for this single- nucleotide polymorphis (SNP).

**Conclusion:**

*ITPA* is likely to be a genetic determinant for decreased fertility. To the best of our knowledge, this is the first report demonstrating this association, however, given the small sample size and other limitations, genotyping of this SNP is recommended to be carried out in different populations with more samples.

## Introduction

Failure to conceive after 12 months of unprotected regular intercourse is commonly defined as infertility (1). A variety of factors may be involved in this process of which genetic factors are perhaps among the best known (2, 3). Also, a number of studies on humans and animal models have suggested that inherited factors may be involved in infertility since the ancestors were said to have been similarly affected (4).

Despite the technological advancement in diagnostic methods, the genetic factors of most infertility cases are not known. Many genetic studies have proposed that different genes might be responsible for male and female infertility (5, 6). Genetic abnormalities, including chromosomal aberrations and single gene mutations are observed in about 15% of male and 10% of female infertile subjects (7).

Oxidative stress (OS) is one of the main factors that may influence fertility due to its role in the modulation of gamete quality and interaction (6, 8, 9). Oocytes, spermatozoa and embryos, and their environments are influenced by free radicals such as reactive oxygen species (ROS) (6). 

Moreover, OS may cause mutations in the DNA molecule. For example, excessive generation of OS may lead to DNA damage in spermatozoa (10). OS and other sources of DNA damage such as reactive nitrogen species (RNS) can affect cellular nucleotides, however, they can be repaired by DNA repair mechanisms. 

Inosine triphosphatase encoded by ITPA is one of the genes that serves as a key sanitizing enzyme of cellular nucleotide pool. The enzyme ITPA catalyzes the hydrolysis of rough purine nucleotides of inosine triphosphate (d/ITP) and xanthine triphosphate (d/XTP) to their monophosphate forms, preventing the accumulation of deaminated nucleotides in DNA and RNA (11, 12).

Different studies have shown the association of *ITPA* deficiency with systemic lupus erythematosus, anemia, adverse reactions to thiopurine compounds, coronary artery disease and other diseases (13-15). Since DNA damage is likely to affect fertility, it is postulated here that *ITPA* deficiency may also be associated with infertility. Sumi et al. (16) showed that patients homozygous for a 94C>A (Pro32Thr, rs1127354) variant display low or absent enzyme activity. 

Based on crystal structure studies, this variant disturbs the affinity for nucleotides and therefore reduces the catalytic activity of *ITPA* (17). Interestingly, this SNP has a high frequency in the Asian population (19%) compared with others (1-7%). We therefore selected this single- nucleotide polymorphis (SNP) to examine its possible association with infertility in the Iranian population. 

Based on previous studies, other polymorphisms such as rs7270101 were identified in this gene which affect *ITPA*activity, by causing alternative splicing and reducing the expression of *ITPA*. The frequency of these SNPs is different in various populations and their association with some diseases have already been shown (18-20). This paper hypothesized that the *ITPA* gene deficiency based on rs7270101 and rs1127354 may be associated with infertility in Iranian patients.

## Materials and Methods

This study was a case-control study of individuals with established infertility. Based on clinical diagnosis, 164 infertile patients (118 females and 41 males) were selected who were referred to the Royan Institute (Infertility Clinic & Reproductive Biomedicine Center, Tehran, Iran) from July 2013 to October 2014. Moreover, 180 ethnically matched fertile controls (132 females and 48 males) were randomly selected from Tehran, Iran. Total genomic DNA was extracted from 500 µl of whole blood using the standard salting out method and stored at -20°C.

Quality and quantity of extracted DNA was evaluated by visualization on 1% agarose gel and spectrophotometry, respectively. The age and sex ratio of cases and controls are presented in Table 1. This study adhered to the Declaration of Helsinki and was approved by Tarbiat Modares University Ethics Committee. Informed written consents were obtained from all participating individuals prior to the sampling. 

**Table 1:** Demographic features of patients and controls


Cases/controls	n	Mean ± SD (age)	Male/Female ratio

Infertile patients	180	31.4 ± 7.9	26.7/73.3
Fertile controls	164	29.5 ± 6.45	25/75


## Genotyping 

For genotyping of two target SNPs we used from mismatch polymerase chain reaction-restriction fragment length polymorphism (PCR-RFLP) strategy. To use this method specific primers were designed by Oligo analyzer software (version 7). For genotyping of: 

rs7270101-F: AAATTGACCGTATGTCTCTGGAATGTTT

and for

rs1127345-F: CAGGTCGTTCAGATTCTAGGAGAAAAGT used as the specific forward primers and a common reverse primer of R: CAAGAAGAGCAAGTGTGGGACAAG used for PCR amplification used as the primers for PCR amplification. The mismatched nucleotides in the forwards primers are presented as underlined. PCR was performed on 50 ng total DNA in a final volume of 20 µl using 10 µl of PCR Master Mix (Solis BioDyne, Estonia) and 4 pM of each primer. The PCR cycling conditions were an initial denaturation at 95°C for 10 minutes, followed by 35 cycles of 95°C for 20 seconds, 60°C for 45 seconds, and 72°C for 45 seconds. After the amplification, the PCR products were digested with the XmnI restriction enzyme (New England BioLabs) according to the manufacturer’s instructions and were run on a 12% polyacrylamide gel (Figes.1, 2). To verify the designed genotyping procedures the DNA sequences of some randomly selected samples for each genotype, was determined by an ABI automated DNA sequencer (Macrogen, Korea). 

**Fig.1:**
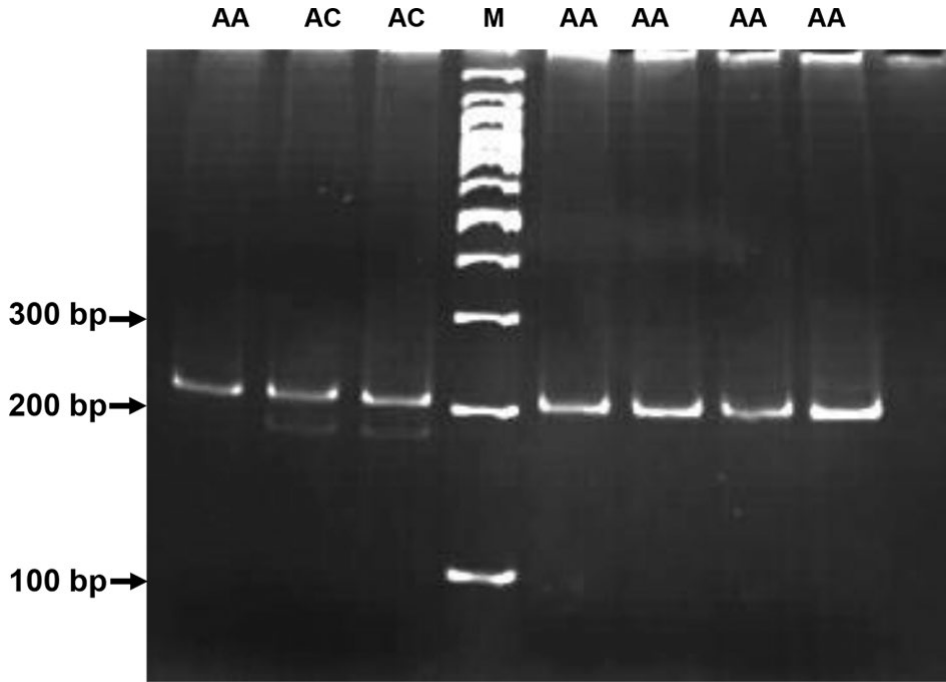
A mismatch PCR-RFLP technique used for genotyping of rs7270101 in *ITPA* gene. The presence of C allele in SNP position can be recognized by XmnI as a restriction endonuclease enzyme. The length of produced amplicon was 204 bp and in digestion process produces 175 bp and 25 bp fragments. The genotype of each sample is shown on top of the gel. ladder is shown by M. PCR-RFLP; Polymerase chain reaction-restriction fragment length polymorphism and SNP; Single-nucleotide polymorphis.

## Statistical analysis 

Genotype frequencies were tested for deviation from Hardy-Weinberg equilibrium (HWE). Moreover, allele and genotype (total, dominant and recessive models) frequencies were compared between the case and control groups by Chi-Square test. Odd’s ratio (OR) and its 95% confidence interval (CI) were obtained to estimate the contribution of the risk factors.

Additionally, a Bonferroni-correction test was carried out to determine the statistical significance level. A P<0.025 was considered significant. All statistical analyses were conducted using the statistical package for the social sciences (SPSS) Version 20 (SPSS Inc., Chicago, IL) and GraphPad Prism 5.

**Fig.2:**
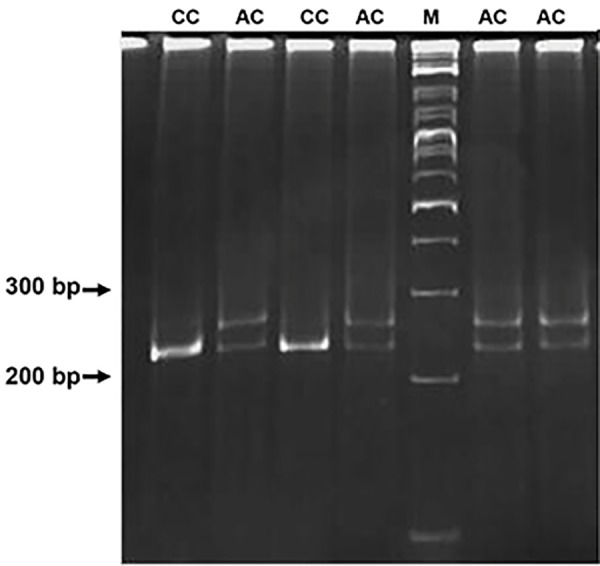
A mismatch PCR-RFLP technique used for genotyping of rs1127354. The presence of C allele in SNP position can be recognized by XmnI as a restriction endonuclease enzyme. Digestion process produces 230 bp and 26 bp fragments. The genotype of each sample is shown on top of the gel. Ladder is shown by M letter. PCR-RFLP; Polymerase chain reaction-restriction fragment length polymorphism and SNP; Single-nucleotide polymorphis.

## Results

Genotype frequencies of both SNPs were in HWE in the control group (P>0.38), however, the genotype distribution of rs1127354 deviated from HWE in patients due to an excess of heterozygotes (P<0.05). Also, a significant difference was found in rs1127354 genotype frequencies between infertile patients and healthy fertile controls (P=0.0001, OR: 2.56, 95% CI=1.86-3.53). Also, based on gender stratification, a significance association was found between this SNP and susceptibility to infertility in male and female groups (P=0.02, OR: 1.8, 95% CI=0.97- 3.349 and P=0.0001, OR: 0.343, 95% CI=0.236-0.49, respectively) (Table 2).

Different genetic models (dominant=CC+AC/AA and recessive=AA+AC/CC) also showed a significant difference between infertile patients and fertile controls (Table 3). 

Contrary to rs1127354, no significant association was discovered between rs7270101 and risk of infertility (P=0.57, OR: 1.73, 95% CI=0.86-2.43). Moreover, the analysis of genotypes in the dominant (AA+AC/CC) (P=0.86, OR: 0.9, 95% CI=0.31-2.6) and recessive (AC+CC/AA) (P=0.57, OR: 1.29, 95% CI=0.61-2.79) models showed no significant association between rs7270101 and infertility. This lack of association was also present at the allelic level (P=0.65, OR: 1.14, 95% CI=0.62-2.1), and after gender stratification in males (P=0.36, OR: 3.6, 95% CI=0.4-33.9) and females (P=0.57, OR: 1.5, 95% CI=0.49-4.8) (Table 4). 

**Table 2:** The genotype and allele distribution of ITPA rs1127354 polymorphism in infertile cases and controls


Rs1127354 Genotype	Cases (%)	Controls	OR (95%CI)	P value	Female cases/Female controls	P value	OR (95%CI)	Male cases/Male controls	P value	OR (95%CI)

AA	34 (20.7)	96 (53.3)	2.56	0.0001	26/74	0.0001	0.343	8.22	0.02	1.8
AC	104(63.4)	74 (41.1)	(1.86-3.53)		76/51		(0.236-0.49)	30.23		(0.97-3.349)
CC	26 (15.9)	10 (5.6)			23/7			3.3		


OR; Odd’s ratio and CI; Confidence interval.

**Table 3:** Association of rs1127354 at allelic, dominant and recessive model levels


Rs1127354	n	Model	P value	OR (95% CI)

	180 control	CC	-	-
	164 case	AC/CC	0.0001	7.34 (3.2-16.8)
		AA/CC	0.126	1.8 (084-4.06)
	Allele	A/C	0.0001	0.39 (0.28-0.53)
	Dominant	AC+CC/AA	0.0001	0.23 (0.140.37)
	Recessive	AA+AC/CC	0.002	3.2 (1.49-6.87)


OR; Odd’s ratio and CI; Confidence interval.

**Table 4:** Association analysis of rs7270101 under different models


Genotype or allele	Infertile number (%)	Healthy number (%)	Analyze model	P value	OR (95% CI)

**AA**	151 (92.1)	162 (90)	Genotype	0.57	1.73 (0.862.43)
**AC**	6 (3.7)	11 (6.1)	Allele A/C	0.65	1.14 (0.62-2.1)
**CC**	7 (4.3)	7 (3.9)	Dominant (AA+AC/CC)	0.86	0.9 (0.31-2.6)
**A**	308 (93.9)	355 (93.06)	Recessive (AC+CC/AA)	0.57	1.29 (0.61-2.79)
**C**	20 (6.1)	25 (6.94)	Female/Female	0.5	1.5 (0.49 -4.8)
			Male/Male	0.36	3.6 (0.43.9)


OR; Odd’s ratio and CI; Confidence interval.

## Discussion

This is the first report which demonstrates this association and therefore should be replicated in other populations. This study was designed based on evidence that OS may cause DNA and nucleotide pool damages. It has been shown that deaminated triphosphate purine nucleotides of d/ITP and XTP can be repaired in an ITPA- dependent manner. It is well known that OS may affect some key properties of sperm and ovum (21), however, no previous study has examined the role of ITPA in infertility. 

We found a significant association between rs1127354 in *ITPA* and infertility under different analysis models. Although the important role of *ITPA* in the genome repair and sanitization of nucleotide pool has been confirmed by different studies, the association of this functional SNP with infertility may shed further light into the molecular mechanism of infertility. Behmanesh et al. (22) demonstrated that Itpa knockout mice (Itpa −/−) die about two weeks after birth with features of growth retardation and cardiac myofiber disarray. In addition, homozygous patients for the 94C>A (Pro32Thr, rs1127354) variant display low or absent enzyme activity (16).

Interestingly, this polymorphism is more common among Asian populations (11-19%) than other ethnic groups such as Africans and Caucasians (1-7%) (13). All these observations suggest that *ITPA* dysfunction may affect the outcome of fertility, which must be considered for further analyses in future molecular studies. The effects of this SNP on *ITPA* activity has been investigated in mercaptopurine metabolism (23), and ribavirin-induced anemia and outcome of therapy in HCV patients (24). Thompson et al. (20) reported that *ITPA* polymorphisms reduce the amount of hemoglobin during treatment with pegylated interferon. The association SNPs and the expression level of *ITPA* has also been assessed in different pathological situations (25, 26).

Low sample size was the main limitation of this study which must be considered in future studies. Interestingly, we found that the case group in this study was not in Hardy- Weinberg equilibrium due to an excess of heterozygotes. This may arise due to a strong association between an allele and disease state, undetected population stratification, genetic mistyping or inadequate sample size. However, given that we observed no deviation in the matched control group, it is most likely due to disease state. Recent studies are concentrated on finding the molecular basis of human disorders and in this way they investigate the role of different molecular aspects of gene regulation. Based on experimental data, certain SNPs in the genome may affect the expression level of genes and therefore are important in their regulation. Non-coding SNPs may increase the susceptibility of disease development by affecting the expression of nearby genes (27). 

The intron 2 SNP rs7270101 is located downstream of the 5’-splice donor site and upstream of the splice acceptor polypyrimidine tract. Anumber of putative consensus branch- site sequences are present in this small 92 bp intron (16) with rs7270101 changing an adenosine nucleotide in one of these sequences, thus possibly resulting in altered expression of *ITPA*. While previous studies examined the role of the DNA repair system in gametogenesis (22), this paper analyzed the association of rs7270101 SNP in the *ITPA* gene with the susceptibility to infertility in the Iranian population.

We observed no association at all levels, however, since no previous study is available on this association, no comparisons were possible. Moreover, all cases and controls were not in HWE for rs7270101, even though controls were randomly selected from ethnically matched people. Undetected population stratification, genotyping errors or inadequate sample size are the main factors for Hardy-Weinberg disequilibrium. In order to check the accuracy of the obtained results, a number of genotyped samples were randomly selected for sequencing and ALL genotypes were confirmed by this method. One possible source of this disequilibrium may be due to the presence of a degree of selective pressure on this SNP, which has been previously observed for immunologically-related SNPs (28, 29). Due to the importance of this functional SNP and the main role of *ITPA* in the DNA repair system, it is recommended that this association is assessed in other populations with larger sample sizes.

## Conclusion

We demonstrate that rs1127354 is associated with infertility under different genetic models and also after gender stratification. Our data is still preliminary and additional studies may help define the actual role of *ITPA* in infertility. Nevertheless, we did not observe this association for the other SNP, rs7270101 with infertility.
